# HIV, Vascular and Aging Injuries in the Brain of Clinically Stable HIV-Infected Adults: A ^1^H MRS Study

**DOI:** 10.1371/journal.pone.0061738

**Published:** 2013-04-19

**Authors:** Lucette A. Cysique, Kirsten Moffat, Danielle M. Moore, Tammy A. Lane, Nicholas W. S. Davies, Andrew Carr, Bruce J. Brew, Caroline Rae

**Affiliations:** 1 University of New South Wales, St. Vincent’s Clinical School, Sydney, Australia; 2 Neuroscience Research Australia, Sydney, Australia; 3 St.Vincent’s Hospital, Neurology and Imaging and HIV Departments, Sydney, Australia; 4 St. Vincent’s Hospital Centre for Applied Medical Research, Sydney, Australia; 5 Department of Neurology, Chelsea and Westminster Hospital, London, United Kingdom; 6 AIDS Dementia and HIV Psychiatry Service (ADAHPS) Sydney Hospital and Sydney Eye Hospital, Sydney, Australia; University of Cape Town, South Africa

## Abstract

**Background:**

Cardiovascular disease (CVD) and premature aging have been hypothesized as new risk factors for HIV associated neurocognitive disorders (HAND) in adults with virally-suppressed HIV infection. Moreover, their significance and relation to more classical HAND biomarkers remain unclear.

**Methods:**

92 HIV− infected (HIV+) adults stable on combined antiretroviral therapy (cART) and 30 age-comparable HIV-negative (HIV−) subjects underwent ^1^H Magnetic Resonance Spectroscopy (MRS) of the frontal white matter (targeting HIV, normal aging or CVD-related neurochemical injury), caudate nucleus (targeting HIV neurochemical injury), and posterior cingulate cortex (targeting normal/pathological aging, CVD-related neurochemical changes). All also underwent standard neuropsychological (NP) testing. CVD risk scores were calculated. HIV disease biomarkers were collected and cerebrospinal fluid (CSF) neuroinflammation biomarkers were obtained in 38 HIV+ individuals.

**Results:**

Relative to HIV− individuals, HIV+ individuals presented mild MRS alterations: in the frontal white matter: lower *N*-Acetyl-Aspartate (NAA) (*p*<.04) and higher *myo*-inositol (mIo) (*p*<.04); in the caudate: lower NAA (*p* = .01); and in the posterior cingulate cortex: higher mIo (*p*<.008– also significant when Holm-Sidak corrected) and higher Choline/NAA (*p*<.04). Regression models showed that an HIV*age interaction was associated with lower frontal white matter NAA. CVD risk factors were associated with lower posterior cingulate cortex and caudate NAA in both groups. Past acute CVD events in the HIV+ group were associated with increased mIo in the posterior cingulate cortex. HIV duration was associated with lower caudate NAA; greater CNS cART penetration was associated with lower mIo in the posterior cingulate cortex and the degree of immune recovery on cART was associated with higher NAA in the frontal white matter. CSF neopterin was associated with higher mIo in the posterior cingulate cortex and frontal white matter.

**Conclusions:**

In chronically HIV+ adults with long-term viral suppression, current CVD risk, past CVD and age are independent factors for neuronal injury and inflammation. This suggests a tripartite model of HIV, CVD and age likely driven by chronic inflammation.

## Introduction

HIV infection is a *chronic* disease. In Australia 95% of treated HIV-infected (HIV+) individuals have a plasma viral load below current detection limits [1]. The HIV epidemic is now characterized by increased life expectancy [2], and a rapidly aging population [3].

New factors for brain injury in virally suppressed individuals are emerging to account for *persisting* HIV-associated neurocognitive disorder (HAND) [4]. Some of these factors have been recently reviewed [5]. The two major candidates are:

1. *Cardiovascular disease (CVD) brain related injury*. A significant body of evidence shows an increased risk of CVD in middle-aged HIV+ individuals as compared to age-matched HIV-negative (HIV−) controls, even when correcting for traditional CVD risk factors [6], [7]. There is emerging evidence that HIV+ adults on Combined Antiretroviral Therapy (cART) have worse neuropsychological (NP) performance partly explained by current CVD [7], [8], [9], but evidence of brain changes at the biochemical or structural level have not been assessed.
2. *Premature brain Aging*. Age-related brain injury may occur at a faster rate in HIV+ individuals than in HIV− age-matched controls. The main evidence comes from MRI-based imaging (see Holt et al., for a recent review [10]). This review identified that potential premature aging preceded the advent of the cART era and was detected in naïve or non-optimally ART-treated HIV+ individuals as increased overall atrophy, perhaps more so in the subcortical areas. But at this time HIV brain involvement was more severe and most individuals were in their 30 s. Therefore, the relevance of these findings to the current context of the HIV epidemic where many are reaching their 50 s and 60 s is limited. It is even more limited when long-term virally controlled HIV+ persons are considered, as age-related factors are likely to play a role (e.g., CVD). In fact, the largest and most recent volumetric MRI study [11], where a high number of participants were virally controlled, found no evidence of an age and HIV interaction, but independent effects in different parts of the brain. Perhaps because atrophic changes already represent an advanced evolution of brain damage, MRI methods more sensitive to changes preceding major neurocognitive deterioration are required. In support of this strategy is a study which has showed cerebral blood flow abnormalities 10–15 years in advance of HIV+ individuals’ chronological age [12]. When concentrating on ^1^H MRS studies (one of the MRI methods of choice to detect sub-clinical biochemical abnormalities in HIV infection) the review [10] found that four have so far assessed the question of HIV and brain aging. All included samples younger than 50 years old on average, potentially limiting the effect of aging per se. All studies included a medium sample size except for the multi-site Neuroimaging HIV consortium study [13] (N = 124 in the HIV+ group), and only two studies included individuals on cART including the largest study [13].All studies included the frontal white matter, frontal grey matter, and the basal ganglia. Two studies included the parietal cortex but no age effect was identified in this region. Overall the most robust finding was that of an increased frontal white matter *myo*-inositol (mIo) and Choline above and beyond what is expected in age-comparable HIV− controls. The effect of CVD was not investigated in any of these studies.

Single voxel ^1^H MRS has several advantages in addressing these issues in HIV infection. Indeed, typical regional moiety alterations in HIV infection in both demented and non-demented patients have been well characterized [14], [15]. There is also evidence that some of the changes are persisting despite long-term cART. The most definitive results on this aspect also come from the large HIV Neuroimaging Consortium study [13]. It showed that there is persistence of those MRS abnormalities in HIV+ adults on cART for a median of 12 years (48% had neurocognitive impairment, and 25% were not virally suppressed). Compared to HIV− controls, they found that mIo and choline (Cho) were abnormally elevated in the frontal white matter in neuro-asymptomatic subjects. *N*-Acetyl-Aspartate (NAA) was reduced in the frontal white matter and basal ganglia in the individuals with HIV-associated dementia (HAD). They did not explore the contribution of CVD but did detect an aging effect as a function of HIV in the frontal white matter for the Glutamate/Glutamine/Creatine (Glx/Cr) ratio and less robustly for the mIo/Cr ratio. Importantly, the authors only included regions traditionally involved in HIV-related brain injury.

^1^H MRS changes in normal aging have also been characterized [16], [17]. In summary, NAA decreases as a function of age in the frontal white matter, while Creatine (Cr) increases. Choline/H_2_O, Cho/NAA and mIo/Cr abnormally increase with age in the posterior brain regions and in the posterior cingulate cortex in particular [17]. However, the extent to which some of those findings are confounded by CVD-mediated brain injury and even brain injury related to Mild Cognitive Impairment (MCI) or early Alzheimer’s (AD) is not clear. Interestingly, the posterior cingulate cortex Cho elevation and NAA decrease show increased abnormalities in individuals transitioning from MCI to AD [18]. Moreover, in middle-aged non-HIV patients with normal cognitive function and diagnosed metabolic syndrome, increased mIo/Cr and Glx/Cr have been found in the occipitoparietal grey matter [19]. In the same brain region and in middle-aged non-HIV individuals with normal cognitive functioning, increased carotid artery intima-media thickness has been associated with decreased NAA independent of age [20].

## Materials and Methods

### Objectives

The current study was designed to address the following two primary aims: 1. Do brain MRS abnormalities in HIV+ adults involve typically affected regions of the brain (frontal white matter and caudate nucleus) or do they extend to regions also affected in pathological aging (e.g., posterior cingulate cortex) in comparison to age-comparable controls? 2. Are CVD and chronological age additional independent neurochemical injury factors in HIV+ adults with virally-suppressed HIV infection?

And two secondary aims: 1. Are immune recovery/activation (plasma CD4+ T cells), HIV duration and inflammatory markers (CSF β2-microglobulin and neopterin) associated with increased neurochemical injury? 2. Which MRS abnormalities are predictive of lower NP performance and how do age, CVD and HIV status may moderate this relationship?

### Participants

Between June 2009 and November 2011, 92 HIV+ individuals and 30 HIV− individuals were enrolled into a prospective study investigating the effects of HIV infection on the brain in middle-aged persons. The HIV+ participants were recruited through the HIV and Neurology outpatient clinics at St Vincent’s Hospital, Sydney. Eligible HIV+ participants: were at least 45 years old; stable HAART for ≥6 months; had a nadir CD4+ lymphocyte count ≤350 cells/mm^3^, and had been HIV-infected ≥5 years. Among those identified (N = 215), 109 (51%) agreed to participate. Three HIV+ women were excluded from the present analyses in order to form a homogenous group regarding known gender effects on MRS major moieties. Four HIV+ men were excluded due to medical history exclusion criteria (one has had a traumatic brain injury with loss of consciousness>30 minutes; one was diagnosed with HIV-related cancer closely after the baseline assessment, one had recent drug abuse, one met criteria for recent alcohol dependence and abuse). Six did not take part in the MRI examination because they had cardiac stents that had not been rated safe at 3T, and three did not participate due to claustrophobia and one elected not to have an MRI.

HIV− controls were recruited via advertising in the metropolitan area of Sydney and across St. Vincent’s Hospital and University of New South Wales campus to target persons of similar demographic and life-style background. To meet eligibility criteria, control participants were required to be HIV-negative on an ELISA test within the past three months. Additionally, because HIV− participants were initially included to provide a local normative and healthy reference for the neuropsychological testing and the ^1^H MRS measurements, individuals with significant or unstable CVD were excluded. More specifically, we excluded individuals with diabetes mellitus, uncontrolled high cholesterol, triglycerides, hypertension, atrial fibrillation, history of myocardial infarction, congestive heart failure, peripheral vascular disease, and carotid/coronary arteriosclerosis.

Exclusion criteria for all participants were: having a history of non-HIV related neurological disorders or psychiatric disorders on axis I (i.e., schizophrenia, bipolar disorder; unstable major depressive disorder); current substance/alcohol use disorders (within 12 months of study enrolment); a history of loss of consciousness greater than 30 minutes; being non-proficient in English; see [21] for details. Participants were not excluded on the basis of current depressive complaints or past alcohol/substance use disorders (predating study entry by 12 months). Recreational use of marijuana was not set as a criterion for exclusion because only severe chronic use has been shown to robustly affect MRS moieties [22]. Furthermore, no participants reported having consumed marijuana in the 12 hours prior to testing. Individuals with hepatitis C (HCV) were included only if successfully treated (HCV RNA-negative).

### Procedures and Investigations

Neurobehavioral evaluation: This evaluation has been described in detail elsewhere [21] (see outline in File S1 and Table S1 in File S1 in Supporting Information S1).

^1^H MRS acquisition and analysis: MR spectra were acquired on a Philips 3T Achieva Quasar Dual imaging system using point-resolved spectroscopy (PRESS) with the following scanning parameters: TE = 31 ms; TR = 2000 ms; number of spectral acquisitions = 64; sampling rate = 2048; 2^nd^ order SHIM, spectral bandwidth = 2000; and phase cycles = 16. Spectra were collected in the following regions of interest: frontal white matter (2.0 cm^3^), posterior cingulate cortex (2.0 cm^3^) and caudate nucleus area (1.5 cm^3^; number of acquisition was set at 64*2 for the caudate area to improve signal to noise ratio – see File S2 in Supporting Information S1 for illustration of voxel positioning). Quantitative analysis of spectra, was completed using jMRUI (Version 3.0; (http://www.mrui.uab.es/). Quantification of the reconstructed signals was performed in the time-domain. All spectra were pre-processed with baseline correction and the Hankel-Lanczos singular value decomposition (HLSVD) function was used to remove the water signal [23]. The AMARES algorithm [24] was used to fit decaying sinusoids, corresponding to Lorentzian line shapes in the frequency domain, to the resonances [25]. Resonances were subjected to soft constraints using a priori input constraining peaks according to frequency range [26]. Fourteen signals were fitted, corresponding to resonance peaks from *myo*-inositol: *N*-acetyl Aspartate (NAA – marker of neuronal density/loss), choline-containing compounds (Cho – marker of cellular membrane turnover), creatine-containing compounds (Cr – marker of cellular energy), glutamine/glutamate (Glx - major excitatory neurotransmitter), aspartate and *myo*-inositol (mIo – marker of neuroinflammation in the context of HIV). All moieties were expressed as ratios with respect to the unsuppressed water signal (H_2_O) and with respect to Cr. We used the H_2_O ratio as it provides a more robust reference than Cr; the latter can vary across the brain even in non-disease states and especially in normal aging [16], [17] (see File S2 in Supporting Information S1 for illustration of typical spectra in each region of interest; additional reference on the recommendation of H_2_O over Cr as a MRS reference and reference for the current MRS protocol). Ratio to Cr was also provided as it is commonly reported in studies using ^1^H MRS in HIV infection [14]. Lastly the posterior cingulate cortex Cho/NAA ratio (to reflect increased in Cho compared to NAA) was included as it is commonly used in aging studies. This ratio is sometimes reported as NAA/Cho (to reflect decrease in NAA compared to Cho) [17].

### Laboratory Visit, Biomarkers Collection and CVD Risk Scores

All HIV+ participants underwent a venepuncture after a 10-hour fast. Consenting HIV+ participants (n = 38) also underwent a lumbar puncture to obtain cerebrospinal fluid (CSF). Blood samples were analyzed at a single laboratory (St. Vincent’s Hospital) for the following: HIV RNA, cholesterol, HDL cholesterol, high-sensitivity C-reactive protein (CRP), CD4+ T-lymphocyte counts. CSF was analyzed for HIV RNA, neopterin and β2-microglobulin. In HIV− participants, fasting cardiovascular-related blood tests and systolic blood pressure results were obtained through their general practitioners as these assays are a routine standard of care across the Sydney metropolitan area.

The 2008 Framingham score [27] was computed to estimate the 10-year CVD risk from age, sex, smoking status, total cholesterol, HDL cholesterol, systolic blood pressure, and current use of blood pressure medication. In addition, the 12-months Data Collection on Adverse events of Anti-HIV Drugs (D.A.D.) CVD score was computed to estimate CVD risk in the HIV+ group [28]. The D.A.D. equation includes the same risk factors similar to the Framingham score, but also current and past use of two protease inhibitors. This score estimates CVD outcomes more accurately than the Framingham score in HIV+ adults [29]. A tonometry examination, as another marker of vascular disease, was also performed in the HIV+ group. Procedure and results for this extra examination are reported in File S3 in Supporting Information S1.

### Ethics

All individuals signed an informed consent before participating in the study and our protocol was approved by the St. Vincent’s Hospital and The University of New South Wales Human Research Ethics Committees. All participants were first assessed with a standard neurobehavioral evaluation. Both the MRI scan and the laboratory examination were conducted within 3 months of the first visit.

### Statistical Methods

#### HIV− versus HIV+ comparisons

To address the first of our primary aims, brain moieties concentration data were visually inspected for the normality of their distributions. Only a few showed mild departure from the normal distribution and we retained the Student’s t-test for our groups’ comparisons as this test remains appropriate in these instances [30]. Those comparisons are presented with and without Holm-Sidak corrections as well as effect size to provide optimal transparency to the reader. The corrections include adjustment for moieties correlation within each voxel (the frontal white matter included 12 comparisons and moieties correlation was r = .35; the posterior cingulate cortex included 10 comparisons and moieties correlation was r = .50; the caudate area included eight comparisons and moieties correlation was r = .25).

**Figure 1:**
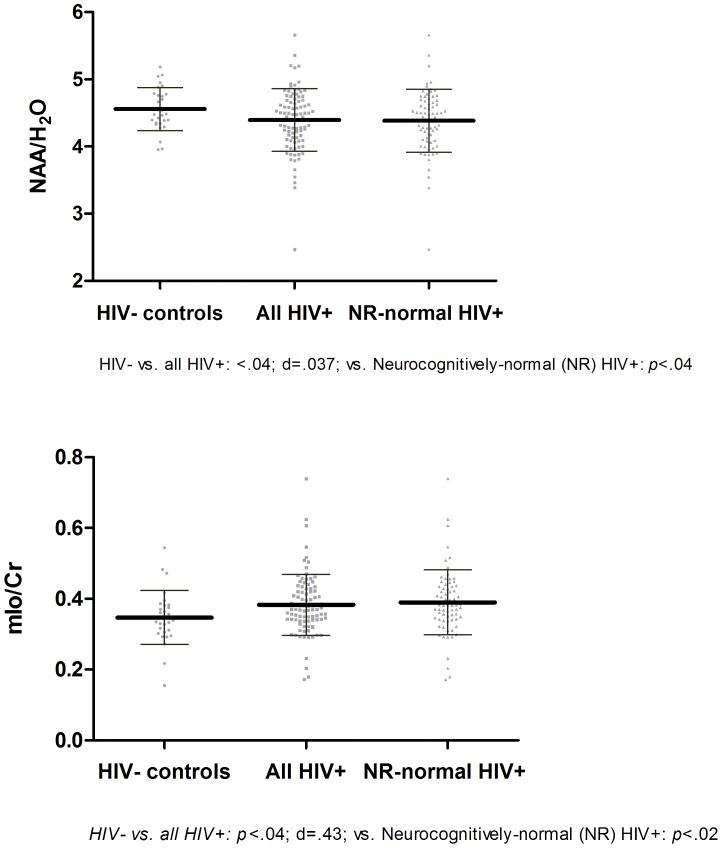
Significant moieties’ difference between the HIV− and HIV+ groups in the Frontal White Matter.

**Figure 2:**
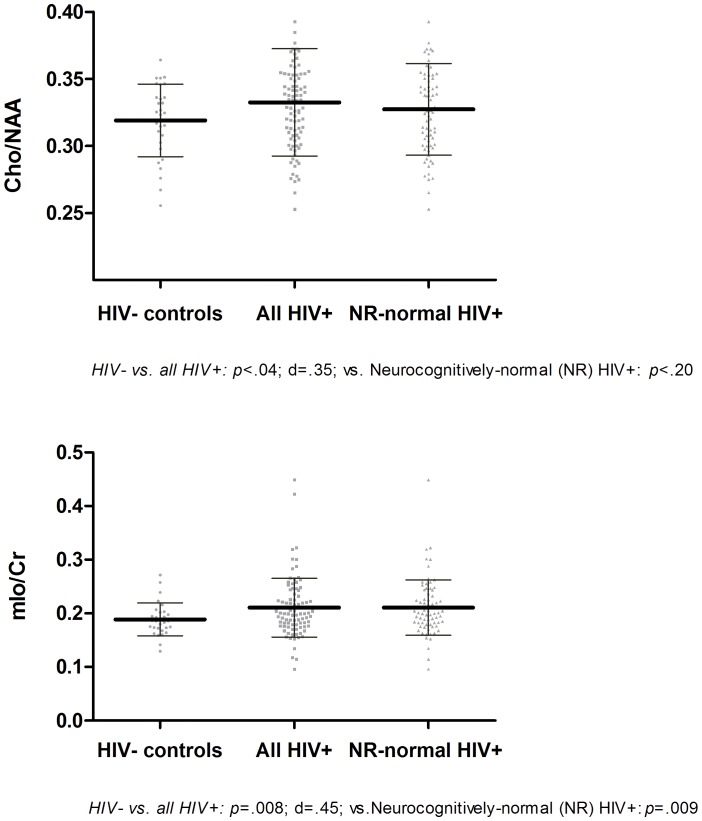
Significant moieties’ difference between the HIV− **and HIV+ groups in the Posterior Cingulate Cortex.**

**Figure 3:**
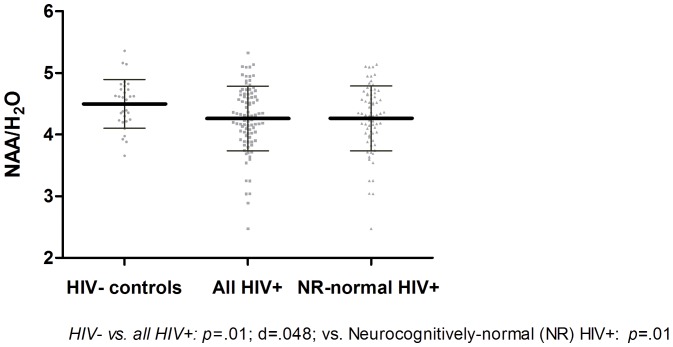
Significant moieties’ difference between the HIV− **and HIV+ groups in the Caudate Nucleus Area.**

Next, to address the second of our primary aims, we conducted stepwise regression models in the entire sample (N = 122) where independent variables were entered in forward fashion and based on whether variables resulted in an improvement in model fit. Model fit was *primarily* assessed using the corrected Akaike Information Criterion (AICc, the lowest AICc yields the best compromise between the model goodness to fit and the model complexity). Model and individual R^2^ and p-values were reported to provide the magnitude of the effects. The models included the moieties that were lower (at *p*<.05; to include medium effect size differences because those may reflect early brain damage, that is brain damage that occur prior to clinical evidence of cognitive deterioration) in the HIV+ group versus the HIV− group, using absolute values or ratio values as appropriate (see Figures 1, 2, 3, and Figures S1, S2 & S3 in File S2 in Supporting Information S1). Models were built separately for each moiety. The Framingham CVD risk score was log_10_ transformed to approximate a normal distribution. From now on we refer to it as “the CVD risk score or CVD”. The models assessed in order: 1. The effect of age: in a first step covariates of “non-interest” were entered in the model in order as follows: (white versus non white) education (years) and then the first covariate of interest: age. 2. The effect of CVD: We added to this initial model the CVD risk score. 3. The effects of CVD and age by HIV status: We added 2-way interaction terms: age*HIV, age*CVD, and CVD*HIV. And, 4. We added a 3-way interaction: age*HIV*CVD to complete the model.

In subsequent analyses and in the regions where a CVD risk score effect was found, we assessed whether the presence of a past acute CVD events added any effect on the MRS CVD-related changes.

Note that HCV was initially entered as a covariate of “non-interest” to check for any effect. It was then excluded so as not to waste the degree of freedom because the concerned numbers were small (3% in each group).

#### HIV biomarkers, CSF and treatment effects and MRS measurements

To address the first of our secondary aims: we conducted a stepwise regression model (same specifications as above) which included the covariates of no-interest, age, CVD and age*CVD interaction and the biomarkers of HIV disease available in all HIV+ participants: nadir CD4+ lymphocyte count, CD4+ T-lymphocyte counts, prior AIDS-defining illness, HIV duration and current cART duration. To assess the D.A.D score, we re-ran the same model replacing the Framingham score with the DAD score. We also tested whether there was an effect of cART as having a greater CNS impact using the CPE-rank score [31]. Finally, the model was applied in the subset with available CSF (N = 38) with the addition of the CSF biomarkers: β2-microglobulin and neopterin.

#### MRS prediction of neuropsychological (NP) performance

To address the last of our secondary aims, we conducted Pearson correlation between the global mean T-score and the nine moieties of interest (see Figures 1, 2, 3, and Figures S1, S2, S3 in File S2 in Supporting Information S1). Then we conducted a stepwise regression model (same specifications as above) with the NP performance as the dependent variable and the moieties as predictors (only those which had a significant effect (*p*<.05) on NP performance in the correlation analyses). Finally the main factors that were significantly associated with MRS measurements in the previous analyses for the retained region (age, CVD, age*CVD, HIV status, CVD*HIV) were included as predictors.

Statistical analyses were conducted using the statistical package JMP 9 (SAS Inc, Cary, NC, USA).

## Results

The demographic and general clinical groups’ characteristics are presented in Table 1; The HIV characteristics are presented in Table 2; and the CVD characteristics are presented in Table 3.

**Table 1:** Demographic characteristics in the HIV+ and HIV− groups.

	HIV− participants	HIV+ participants	*P*
N	30	92	
Age (years)	54.30 (6.41)	55.70 (7.50)	>.31
Age range	45–67	45–74	–
Age >60 years old	27%	31%	>.61
Education (years)	15.36 (2.71)	13.96 (2.91)	<.05
Gender (% Male)	100%	100%	–
Handedness (%Right)	90%	89%	>.90
% Caucasian	97%	92%	>.67
FSIQ	114.58 (7.86)	111.40 (10.14)	<.10
HIV Risk groups (%MSM)	90%	88%	>.90
Currently Employed	58%	54%	>.68
% HCV+	3%	3%	>.98

Mean SD otherwise notified; MSM: Men who have sex with men; FSIQ: Predicted Full scale IQ as determined by the NART. Ethnicity: In control group –1 Asian-Australian; 1 Middle-Eastern/Mediterranean Australian In HIV+ group: 1 Asian-Australia, 1 Indigenous-Asian Australian, 2 Middle-Eastern/Mediterranean Australian, 1 South American Australian. All but one had secondary education in English, and this participant had secondary education in Spanish and had lived in Australia for 30 years. HCV: Hepatitis C Virus (Note that all HCV+ individuals were not HCV active as per criteria of inclusion/exclusion).

**Table 2:** HIV disease, laboratory and HAART characteristics in the HIV+ group.

Characteristics in participants with HIV infection	Measurements
Estimated HIV duration (Median years, Min-Max)	20 (5–30)
% AIDS	72%
% AIDS Defining Illness	45%
Nadir CD4 (cells/mL, Median, IQR)	180 (52–287)
Current blood CD4 (cells/mL, Median, IQR)	527 (342–720)
Current blood CD8-T cell count (cells/mL, Median, IQR)	822 (628–1150)
% Plasma HIV RNA (undetectable)	98%
% CSF HIV RNA (undetectable)^1^	97%
CSF Neopterin (mmol/L)	13.77 (4.64)
CSF Beta-2 microglobulin (mg/L)	1.53 (0.40)
Current treatment duration in months (Median years, Min-Max)	24 (6–156)
% On a high CPE (>7)^2^	78%
% High Adherence^3^	94%

1 N = 38 who had CSF lumbar puncture.

2 Central Nervous System Penetration Efficiency (CPE) rank score definition [40].

3 High antiretroviral adherence: percentage of participants who reported taking >95% of their antiretroviral medication doses over the past seven days.

IQR: Inter-quartile range.

Undetectable: HIV RNA assay with a limit of detection at 50 copies/mL.

**Table 3:** Cardio-vascular risk factors, treatment and disease characteristics in the HIV− and HIV+ groups.

	HIV− participants	HIV+ participants	*P*
2008 Framingham CVD risk (Median, Min-Max)	8.0 (3.5–31.9) [>15 = 23%]	15.0 (4.3–79.7) [>15 = 53%]	.0006
12-month risk CVD D.A.D. (Median, Min-Max)	–	1.01 (0–5.7)	–
Tonometry Augmentation Index (Median, Min-Max)	–	19.92 (2–46) [>22 = 41%]	–
Total Cholesterol (mmol/L)	5.6 (1.4)	5.03 (1.12)	.22
HDL Cholesterol (mmol/L)	1.46 (0.21)	1.21 (0.36)	.004
Hs CRP (mg/L) (Median, Min-Max)	–	1.6 (0.1–117) [>10 = 10%]	–
Systolic blood pressure (mm/Hg)	121.7 (16.6)	130.3 (15.1)	.12
% History of Diabetes	0%	5%	.19
% Past CVD major event^1^	0%	16%	<.02
% Currently Smoking	10%	19%	.22
% Ever smoked	30%	42%	.24
% On anti-hypertensive medication	23%	23%	.97
% On anti-lipid lowering medication	13%	31%	.05
% On Aspirin/anti-coagulants/anti-platelets	0%	12%	.05
% On Abacavir^2^	–	37%	–
% Ever been on Lopinavir/Ritonavir^2^	–	39%	–
% Ever been on Indinavir^2^	–	37%	–

1 Atrial fibrillation, Myocardial infarction, congestive heart failure, peripheral arteriosclerosis, carotid/coronary arteriosclerosis.

2 This information was specifically part of the D.A.D. score and was entered as “yes” vs “no” for Abacavir and as the duration in months for Lopinavir/Ritonavir and Indinavir.

Note that there was a strong correlation between the Framingham score and the D.A.D. score in the HIV+ group (r = .67; p<.0001). There was moderate correlations between the Framingham score and the Augmentation Index (*r* = .31; *p*<.005) and the D.A.D. score and the Augmentation Index (*r* = .38; *p*<.004).

Standard assays for Cholesterol, Reference Intervals: 0.0–6.0 mmol/L; Auto Chemistry (8382–9164).

Standard assays for HDL Cholesterol, Reference Intervals: >1.0 mmol/L; Auto Chemistry (8382–9164).

Standard assays for High-sensitivity C Reactive Protein(hs-CRP), Reference Intervals: <10 mg/L; Auto Immunoassay (8382–9161).

### ^1^H MRS Brain Moieties in Study Groups

In the frontal white matter (Figure 1 & Figure S1 in File S2 in Supporting Information S1), the NAA/H_2_O was lower (*p* = .0371; but not-significant with Holm-Sidak cut-off *p*<.0101) in the HIV+ group and the ratio of mIo/Cr was higher (*p* = .0372; but not-significant Holm-Sidak cut-off *p*<.0107) in the HIV+ group, driven by a lower Cr/H_2_O and higher mIo/H_2_O in the HIV+ group. The mIo/Cr yielded a medium between-groups effect size; while the NAA yielded a small to medium effect size. In the posterior cingulate cortex (Figure 2 & Figure S2 in File S2 in Supporting Information S1), the ratio of mIo/Cr was elevated (*p* = .0079; and significant with Holm-Sidak cut-off *p*<.0160) and consisted mostly of elevated mIo/H_2_O. Moreover, Cho/NAA ratio was elevated (*p* = .0412; but not-significant with Holm-Sidak cut-off *p*<.0169) in the HIV+ group driven mostly by a lower NAA/H_2_O. The magnitude of the effect size for the mIo/Cr ratio was in the medium range; while the magnitude of the effect size for the NAA/Cho ranged in the small to medium range. In the caudate nucleus area (Figure 3 & Figure S3 in File S2 in Supporting Information S1), NAA/H_2_O was found to be lower (*p* = .0113; but just above the significant Holm-Sidak cut-off *p*<.0107) in the HIV+ group yielding a medium effect size. Comparisons between neuropsychologically normal HIV+ (N = 71) and normal HIV− (N = 29) participants yielded similarly different results in all regions and all moieties except for the posterior cingulate cortex where the difference for the Cho/NAA ratio was no different (*p* = .20), see Figure 1, 2 & 3.

### The Effect of Age on Brain Moieties

Based on the AICc we found that age was associated with decreased NAA/H_2_O in the frontal white matter (age & best model R^2^ = .030; *p* = 0.06; AICc = 144.6) and decreased NAA/H_2_O in the posterior cingulate cortex (best model R^2^ = .040; *p*<0.03; AICc = 202.1). Of note these models also included ethnicity and education as covariate of “no-interest”. Only higher education was found to be associated with increased NAA/H_2_O in the caudate nucleus area (education R^2^ = .052; *p*<0.02; AICc = 177.1). In this model age was also marginally associated with decreased NAA/H_2_O (*p*<.08) and improved the overall model (age & education, best model R^2^ = .076; AICc = 176.1).

### The Effect of CVD on Brain Moieties

Based on the AICc and when age and the CVD score were now included in the models, we found that a higher CVD risk score was associated with decreased NAA/H_2_O in the posterior cingulate cortex (CVD & best model R^2^ = .11; *p* = 0.0002; AICc = 192.3). Also a higher CVD risk score was marginally associated with an abnormally elevated Cho/NAA (CVD & best model R^2^ = .05; *p*<.09; AICc = −449.4) as well increased mIo/Cr (CVD R^2^ = .05; *p*<.02; AICc = −379.5) in the posterior cingulate cortex. Lastly, a higher CVD risk score was associated with decreased NAA/H_2_O in the caudate nucleus area (CVD R^2^ = .06; *p*<.007; AICc = 176.3). Age was not an AICc retained predictor in those models, with the exception of the frontal white matter lower NAA/H_2_O effect that was previously detected.

### Age & CVD & HIV Status Effects on Brain Moieties

Then we included the HIV status, as well as its interactions with age (HIV*age) and CVD (HIV*CVD) and age*CVD interaction term in the models. Based on the AICc, we found a significant age*HIV interaction effect in the frontal white matter. Namely decreased NAA/H_2_O was associated with older age and more so in the HIV+ group than in the HIV− group (interaction & best model R^2^ = .095; *p* = .009; AICC = 140.5). The addition of the 3-way interaction (age*HIV*CVD) did not add any explanatory power to the regression models and was therefore left out in the subsequent analyses.

### Age & CVD & HIV Status & Past CVD Acute Events Effects on Brain Moieties

**Figure 4:**
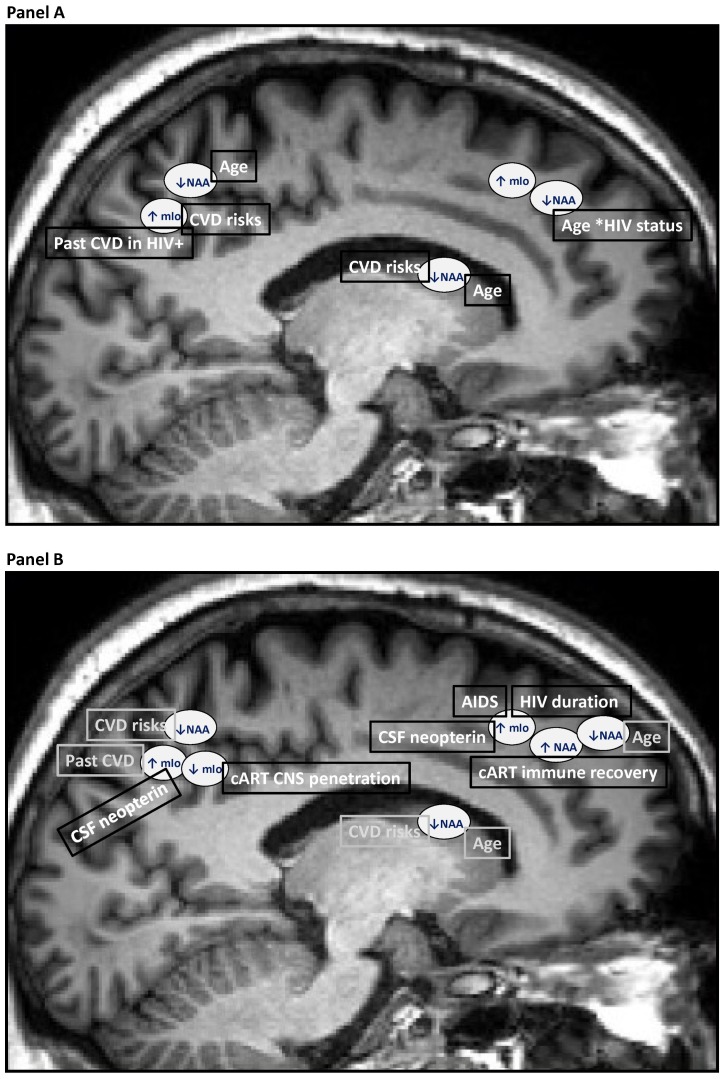
Age, CVD and HIV status predictors of brain moieties changes in the entire sample (panel A); Age, CVD, HIV biomarkers predictors of brain moieties changes in the HIV+ sample (panel B).

To determine if in addition to the current CVD risk score, past acute CVD may have a role in current brain moieties changes where a CVD risk score effect had an effect (i.e., the posterior cingulate cortex and the caudate nucleus area), we first added the acute CVD data in the model (16% of the HIV+ group had had previous acute CVD events versus none per design in HIV− group (see Table 3)). We found that in the posterior cingulate, there was a marginal effect of past acute CVD events on decreased NAA/H_2_O (*p*<.14) and slightly improving the overall model in addition the CVD risk score (best model R^2^ = .0115, AICc = 192). There was also a significant effect of past acute CVD events leading to an increased mIo/H_2_O in the same region (R^2^ = .04, *p* = .03, AICc = −45.5). In the caudate nucleus area, there was a marginal effect of past acute CVD event (*p* = .13) on decreased NAA/H_2_O slightly improving the overall model (best model R^2^ = .012, AICc = 172.6). The addition of an interaction term (CVD*CVD past acute event) did not add any explanatory power to the models. See figure 4A which illustrates and summarizes the findings based on the stepwise regression models in the entire sample (N = 122).

### HIV & CSF Biomarkers and Metabolites Concentrations in the HIV+ Group

Immune recovery: Based on the AICc, greater immune recovery (defined as current CD4 mines nadir CD4) (*p*<.07) yielded a marginal effect on increased NAA/H_2_O in the frontal white matter, but the negative age effect (*p*<.02) remained the main factor (age & immune recovery R^2^ = .10; AICc = 116.9).

HIV duration: In the frontal white matter, a longer HIV duration was marginally associated with increased mIo/H_2_O (R^2^ = .04; *p* = .06; AICc = 6.3) as well as decreased NAA/H_2_O (AICc = 116.8; *p* = .12). In the posterior cingulate, longer HIV duration was marginally associated with lower NAA/H_2_O (AICc = 152.6; *p*<.09), yet improving the overall model in addition to the CVD risk score and the presence of past acute event (best model R^2^ = .017; AICc = 152.2). Lastly, in the caudate nucleus area a longer HIV duration was a significant factor for decreased NAA/H_2_O (AICc = 139.4; *p* = .003) and this yielded the best model with the effect of CVD risk score previously described (best model R^2^ = .010; AICc = 139).

AIDS defining illnesses: A greater number of past AIDS defining illnesses was associated with increased Cr/H_2_O (R^2^ = .06; *p*<.02; AICc = 56.9), increased mIo ((R^2^ = .09; *p* = .02; AICc = 2.9) in the frontal white matter.

The D.A.D score: As for the Framingham score, the 12-months CVD risk score was associated with decreased NAA/H_2_O in the posterior cingulate (R^2^ = 0.11, p<.0001; AICc = 154.3). There were no other associations.

CSF biomarkers (models conducted in the 38 HIV+ participants with available CSF data): Based on the AICc, having a CSF neopterin >13 nmol was associated with increased mIo/H_2_O marginally in the frontal white matter (*p* = .09; AICc = −3.1) and in the posterior cingulate (*p* = .10; AICc = 5.6).

### Greater cART CNS Penetration Effect on Moieties Concentrations in the HIV+ Group

We found that a CPE rank score >7 was associated with increased NAA/H_2_O (marginally so after the effect negative impact of the CVD risk score and longer HIV duration; R^2^ change = .0025, *p*<.10, AICc = 152) and decreased mIo/Cr (R^2^ = .004, *p*<.04, AICc = −269.3) in the posterior cingulate cortex. See figure 4B which illustrates and summarizes the findings based on the stepwise regression models in the entire HIV+ sample (N = 92) the sub-sample with CSF data (N = 38).

### Neurocognitive and Mood and Functional Characteristics in Study Groups

The detailed results are reported in File S3 and Table S2 in File S3 in Supporting Information S1. In summary The HIV+ group showed significantly more overall neurocognitive impairment (*p*<.002) and 22% of HIV+ participants were classified as HAND compared to 3% in the HIV− group (*p*<.02).

### MRS Measurements Predictions of NP Performance

The only correlation between MRS measurements and NP performance was for the Cho/NAA when all subjects (N = 122) were included. In the regression model, Cho/NAA was abnormally elevated as a function of lower NP performance and more so in the HIV+ group than the HIV− group (Interaction and best model R^2^ = .012; p = .002; AICc = 733.3). No other factor made an impact on the model.

## Discussion

Our study is characterized by several findings which may represent some aspects of a new model of CNS injury in chronic and virally controlled HIV infection. Current CVD risk, past acute CVD events and age are independently associated with a mild degree of neuronal injury and a clinically relevant degree of neuroinflammation. Further, our results point to additional factors such as HIV duration, greater CNS cART penetration and the degree of immune recovery on cART. This suggests a tripartite model where HIV, CVD and age lead to chronic CNS inflammation and early neuronal damage. That is damage that precedes major neurocognitive deterioration. Indeed, this cohort is composed of a majority of stable HIV+ individuals with relatively low neurocognitive dysfunction (HAND = 22%), although this prevalence is in line with what has been reported in the cART era in similar types of HIV+ individuals [4].

The mild neurochemical alterations involved classically affected regions (frontal white matter and caudate) [12]–[15] as well as a new region, the posterior cingulate cortex even with normal neurocognitive functioning. In all instances the magnitudes of the moieties’ differences relative to controls were within the small to medium range (d = .30–.45). This degree of change is plausible in a cohort that is optimally treated, with a relatively low rate of HAND and importantly with no or low psychiatric pre-morbid confounds or co-morbidities. Indeed, in previous cART studies which have been all conducted in the USA (see [10]), where HAND is still unfortunately driven by a substantial number who do not adhere to cART and uncontrolled viral load, the differences between HIV− and HIV+ in terms of MRS abnormalities have been found to be within the medium to large range and quite consistently involving Cho. This is for example evident when including at least half of the sample with HAND [13]. In Australia, the HIV epidemic characteristics are different due to a combined effort of the HIV physicians on the adherence to cART. Among the Western world Australia has one of the highest rates of viral suppression at a national level meaning that our cohort is representative [1]. Still our cohort is composed of many survivors from the pre-cART era and some of those may be particularly robust individuals in relation to the effect of HIV and brain aging. We reported both Holm-Sidak corrected and non-corrected results. Interestingly when doing so, one of the most consistent differences in HIV studies (i.e., one of the basal ganglia nuclei) fails to reach the corrected significant cut-off despite having a relative largest effect size in the current study. This suggests that the Holm-Sidak correction may lead to Type II errors in the context of an optimally treated cohort. We further discuss in the limitations’ section the problem of using such corrections when the effect size to be detected is small to moderate, yet of possible clinical consequence.

### Neurochemical Change Related to HIV

Neuronal dysfunction of small to moderate severity was present in both the frontal white matter and the caudate nucleus area despite plasma and CSF HIV viral suppression. The presence of lower NAA and increased mIo provides evidence for both chronic neuroinflammation and neuronal injury, which is consistent with findings of cART era persistence of brain metabolic abnormalities in HIV+ adults, although here the magnitude of the neuronal injury is less than previously reported [10], [13]. It is not clear whether such chronic neuroinflammation is driven by low level (below standard detection techniques at least in the CSF) HIV replication or other processes [32]. Moreover, there was an increased HIV duration effect on lower NAA concentration more so in a region known to be susceptible to primary HIV injury burden (i.e., caudate), and independently of the age factor. Also, immune recovery was associated with higher NAA levels in the frontal white matter suggesting that long-term recovery continues to play a role in current brain neurochemical repair. In parallel, CSF neopterin was linked to increased cellular activation and inflammation in the frontal white matter and in the posterior cingulate cortex suggesting this process may happen across the brain. Recent MRS findings in the macaque model of NeuroAIDS [33] add credence to our findings. In this animal model NAA was associated with actual neuronal injury using immunohistochemistry and stereology measures to show decreased synaptophysin, and microtubule-associated protein. They also found that elevated Cr may reflect enhanced high-energy phosphate turnover in highly metabolizing activated astrocytes and microglia. The association we found between increased Cr levels in this region and neopterin, which is a persistent immunoactivation marker in chronic HIV infection [34], would confirm a model of chronic immune activation compounded by disease duration.

We found that the CPE-rank score was associated with a mild reduction of mIo in the posterior cingulate mainly. This is the first study to identify such an effect and in this region of the brain. Interestingly our cohort was composed of a high percentage of individuals on a higher CPE (>7) regimen and it is conceivable that a larger effect may have been found in a cohort less optimally treated on this aspect as well. The CPE score effect remains controversial despite some real possibility for its effect [35]. Our study seems to recommend that if a clinical trial is conducted the inclusion of MRS to assess sub-clinical effect in addition to NP method would be an asset.

### Neurochemical Change Related to Age

Consistent with and extending previous findings in middle-aged cohorts [13], we found that age and chronic HIV infection interact, leading to neuronal dysfunction in the frontal white matter, adding further evidence to the possibility of accelerated neuropathological aging. Importantly, we demonstrated that this effect was observed even when CVD was accounted for in the regression models. The HIV Neuroimaging Consortium study, also conducted in chronic patients, found a steeper effect of age for Glx and mIo, but not for NAA. These differences in the moieties involved are likely to be related to the high level of clinical stability and viral control (2% versus 25%) in our cohort and a lower level of clinically relevant neurocognitive impairment (22% versus 48% of impairment). The fact that the frontal white matter is involved in an HIV interacting age effect (rather than either of the two other regions studied) is interesting and adds credence to the possibility of an accelerated brain process, as the frontal white matter has been robustly and primarily implicated in normal aging, including white matter biochemical changes [36].

The finding of chronic neuroinflammation and neuronal injury to a lesser extent in the posterior cingulate is novel. This specific region has not been previously investigated in middle-aged HIV+ individuals. Although the parietal cortex has been evaluated in pre-cART studies, these studies included patients with severe dementia in whom the whole brain was affected [37]. Cho/NAA and mIo have been found to be altered, in this region, in patients with AD [38] and patients converting from MCI to AD [18]. In addition, an abnormal Cho/NAA ratio was predictive of lower global NP performance rather than any moieties in regions typically targeted by HIV. This suggests that some of the current NP impairment is associated with cortical injury even in those with optimal treatment. The functional impact of these neurochemical changes remains unclear as there were no direct associations with decline in activities of daily living (analyses not shown). However, the latter was associated with worse NP performance (see File S3 in Supporting Information S1).

### Neurochemical Change Related to CVD

CVD risks lead to increased neuroinflammation and neuronal injury in the posterior cingulate cortex and neuronal injury in the caudate nucleus area for both the HIV+ and HIV− participants However, there was no statistical evidence that this was a stronger effect in the HIV+ group. This despite the fact that the HIV+ group was significantly more at risk for a 10-year CVD event as predicted by the Framingham and may be partly explained by ongoing brain tissue repair associated with optimal cART and the high level CPE in this cohort. This finding warrants caution even though there was no evidence that the HIV+ patients have more current CVD-related brain injury. This is for two reasons; first, these abnormalities are consistent with ^1^H MRS abnormalities found in patients with metabolic syndrome [19], and patients with sub-clinical arteriosclerosis [20]. Second, we found that past acute CVD events were related to MRS abnormalities and mainly to greater mIo concentration in the posterior cingulate cortex. In other words, those at risk now may develop greater CVD-related injury as they develop an acute event (this is known to happen despite being on treatment [39]), and possibly irreversibly as our findings would suggest. The cross-sectional nature of our analyses cannot fully determine how much of an impact this may have. Longitudinal analyses will be crucial in both age-matched HIV− individuals and ideally including some with CVD burden as well. Other reference groups may be in fact chronic CVD patients for which ^1^H MRS is just emerging as noted above.

The D.A.D. score did not yield a higher explanatory power compared to the Framingham score in the HIV+ cohort. The fact the prediction is limited to12 months could be a factor. Another possibility is that the inclusion of the potentially cardio-toxic effect did not in fact impact MRS measurements. The question remains open for a longitudinal study.

### Limitations

We conducted 30 initial between-group independent comparisons (Students’t-test) for the brain moieties in selected regions of interest (as opposed to any whole-brain analyses). In the traditional framework of *experimentwise error rate*, this corresponds to 1.5 expected false-negative at *p*≤.05. We also provided Holm-Sidak corrections, but those do not apply optimally to our study design. Indeed, using the stricter p-value cut-off, the caudate finding, which is probably one of the most robust findings in the HIV MRS literature, was not significant despite taking into account the within voxel moieties correlation. Experiment-wise corrections have many intrinsic limitations and this is why they are strictly required only for confirmatory studies, most often in the context of clinical trials [40]. It is possible that only with a much larger sample size, some of our p-values would have become more significant in this optimally treated cohort. Because, there in fact is no agreement in the statistical community as to whether multiple comparisons are a correct analytical choice, transparency in the statistical plan and a clear rationale in the statistical choices are advocated and this is the strategy we have followed [40].

At outlined above our HIV+ sample represents the specifics of the Australian epidemic with a high level of viral suppression and stable HIV risk factors. This means that our cohort is composed in majority of white gay men with high pre-morbid functioning and derived from tertiary healthcare urban hospital. Therefore our findings may lack generalizibility especially for women and more vulnerable HIV+ groups (drugs users, adults from poor socio-economic background, adults from some ethnic minorities) who may in fact be at greater risk of brain CVD and aging-related damages than our current cohort.

## Supporting Information

Supporting Information S1 **Supporting Files.**
(PDF)
