# Supplementary material for: HIV, Vascular and Aging Injuries in the Brain of Clinically Stable HIV-Infected Adults: A 1H MRS Study
Source: PLoS One. 2013 Apr 19;8(4):e61738. doi: 10.1371/journal.pone.0061738 (PMC3631163; doi:10.1371/journal.pone.0061738)
Supplement: Supporting Information S1 — Supporting Files. (PDF) [file pone.0061738.s001.pdf]

Supplemental files contents	
**File S1**	Page2
Standard neurobehavioral evaluation	Page 2
Table S1	Page 4
**File S2**	
Localization of the voxel for brain spectra acquisition	Page 5
Rationale for voxel selection	Page 6
Rationale for unsuppressed water signal (H_2O) as the main reference	Page 6
Spectra illustrations	Page8
Figures S1, S2 & S3	Page 11-13
**File S3**	Page 14
Tonometry examination procedure	Page 15
Extra results section	Page 15
Table S2	Page 16
References	Page 17

File S1

Standard neurobehavioral evaluation

Prior to the assessment of neurocognitive functions, information regarding participants' medical, social and occupational history was obtained through a basic demographic inventory, administered via an interview format. Depressive complaints, cognitive complaints, and Independence in Activities of Daily Living (IADL) were assessed with standard self-report instruments (Beck Depression Inventory-II [1,2], Personal Assessment of Own Functioning Inventory[3] and IADL [4]). Antiretroviral medication adherence was also recorded using a questionnaire was adapted from [5] recommendations. The neurobehavioral examination also included a brief neuropsychiatric semi-structured interview (Electronic Mini International Neuropsychiatric Interview - eM.I.N.I version 5.2 [6]). On this examination, we found that 11% of the HIV+ participant had experienced a major depressive episode. As per design, no participants were found to have current substance abuse or dependence disorders. All participants underwent a standard neuropsychological evaluation to assess functions of attention/working memory; verbal learning and memory, verbal generativity; fine-motor coordination; mental flexibility/inhibition and speed of information processing (See Table S1 for individual tests). The session duration was approximately two hours.

The standard neuropsychological testing was conducted by research assistants/students who were neuro/psychology graduates (EF, TL and DM were trained in the administration of the testing protocol under the supervision of a senior neuropsychologist, LAC). All measures were administered and scored according to standard procedures. Neuropsychological scores were

converted into demographically corrected T-scores and deficit scores to compute overall impairment rate and domain specific performance scores (Heaton et al., 2004; Carey et al., 2004). Using published normative data [7,8], raw scores were converted to demographically corrected T-scores in order to minimize the influence as appropriate of age, education, sex and ethnicity (Caucasians versus other). T-scores were then transformed into a Global Deficit Score (GDS,[9]) according to the following criteria: *T*-scores greater than 40 reflects no impairment (deficit score = 0), whereas a deficit score of 1 reflects mild impairment (*T* score = 39 to 35), deficit score of 2 reflects mild to moderate impairment (*T* score = 34 to 30), 3 reflects moderate impairment (*T* score = 29 to 25), 4 reflects moderate to severe impairment (*T* score = 24 to 20), and 5 reflects severe impairment (*T* score <20). Deficit scores on all tests were averaged to create the GDS. A GDS ≥ 0.5 a clinically validated and reliable cutoff [9] which indicates that, on average, an individual is at least mildly impaired in at least half the single neuropsychological measure in the battery (the current battery was composed of 16 neuropsychological measures).

Table S1: instruments included in the standard neurobehavioral assessment

DOMAINS	*STANDARD MEASURES*
DEPRESSIVE & COGNITIVE COMPLAINTS	*Beck Depression Inventory –II*: assesses the existence and severity of symptoms of depression
	Instrumental Activities of Daily Living Scale: this is a modified version of the Lawton and Brody scales, which measure the functional impact of emotional, cognitive, and physical impairments
	Patients Assessment of Own Functioning Inventory: intended to evaluate a patient's sense of functional capacity in everyday
ADHERENCE	*Antiretroviral Medications Adherence Questionnaire (AMAQ)***:** Assesses the patient's adherence to antiretroviral medications over the past week. It also assesses what is the most common reason a patient misses a dose of their medication
PSYCHIATRIC INTERVIEW	*Electronic Mini International Neuropsychiatric Interview* (eM.I.N.I)*:* a brief neuropsychiatric semi-structured interview used to assess the presence and severity of DSM-IV and ICD-10 psychiatric disorders
PRE-MORBID ABILITIES	*National Adult Reading Test- Revised:* A reading test consisting of 50 irregularly spelled words. This test was omitted in the six participants with English as a second language to avoid incorrect pre-morbid ability estimation.
MOTOR FUNCTIONS	*Grooved Pegboard*: A test of manipulative dexterity [10]
VERBAL LEARNING & MEMORY	*Hopkins Verbal Learning Test –Revised*: A brief learning and memory test [7]
ATTENTION/WORKING MEMORY	*WAIS-III Letter-Number Sequencing*: Involves ordering, in numerical and alphabetical order, numbers and letters that have been presented verbally in an unordered sequence [8,11]
	WMS-III Spatial Span: Requires the participant to tap a series of blocks in the same order as the examiner and in the reverse order of what the examiner touched [8,12]
SPEED OF INFORMATION PROCESSING & MENTAL FLEXIBILITY & INHIBITION	*Trail Making Test A and B*: The participant's task is to draw lines that connect 25 numbered circles in ascending order. In part B, there is the added task of switching between numbers and letters [8]
	Digit Symbol Coding: Requires the participant to fill the blank spaces according to a key for 120 seconds [8,11]
	Colour-Word Interference Test: A speed-based test with four conditions that require the participant to name ink colours, read words and inhibit prepotent responses [13]
VERBAL GENERATIVITY	*Controlled Oral Word Association Test:* A measure of executive functioning which evaluates the spontaneous production of words within one minute [8,14]

File S2

Localization of the voxel for brain spectra acquisition

Frontal White Matter Spectroscopy: Voxel was positioned in the Right Frontal White Matter, while avoiding as much grey matter as possible.

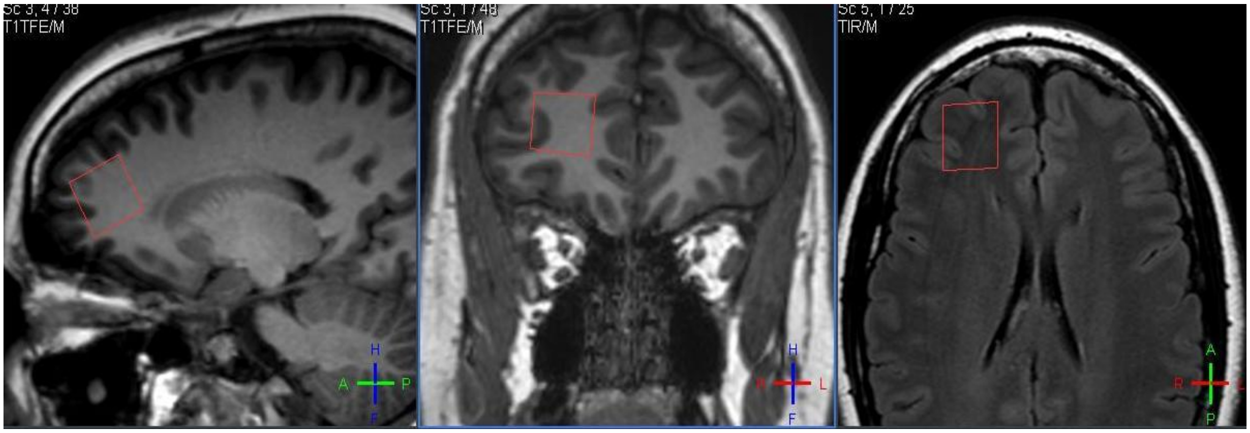

Posterior Cingulate Spectroscopy: This ROI spanned the mideline to include right and left posterior cingulate gyri and inferior precunei. The superior border of corpus callosum and the cingulate sulcus were the anatomical landmarks to define the anterior inferior and the anterior superior border of the voxel. No axis manipulation was required.

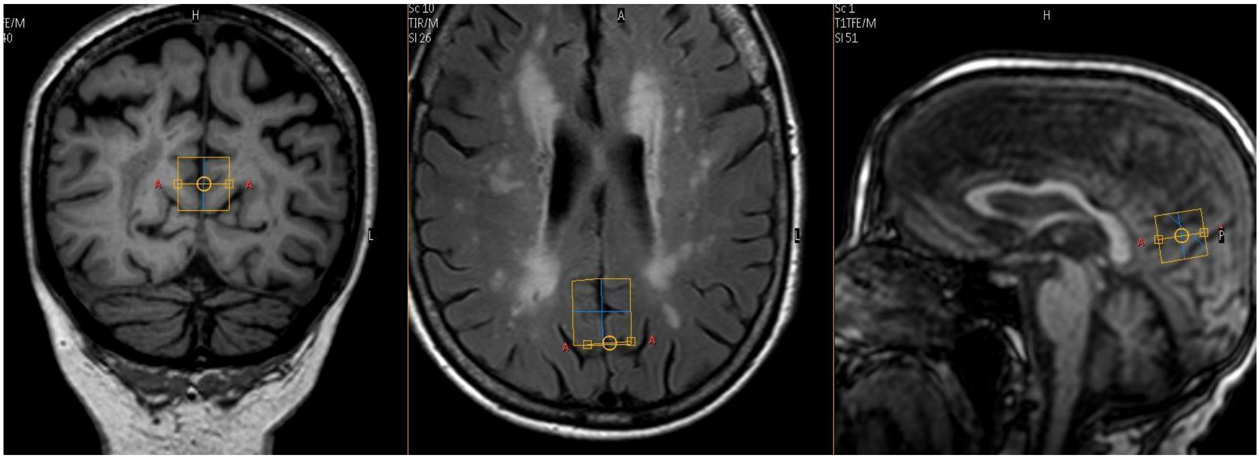

Right Caudate Nucleus / Basal ganglia

Voxel was positioned as much as possible within the right caudate nucleus. When severe atrophy was present the voxel was positioned in the basal ganglia.

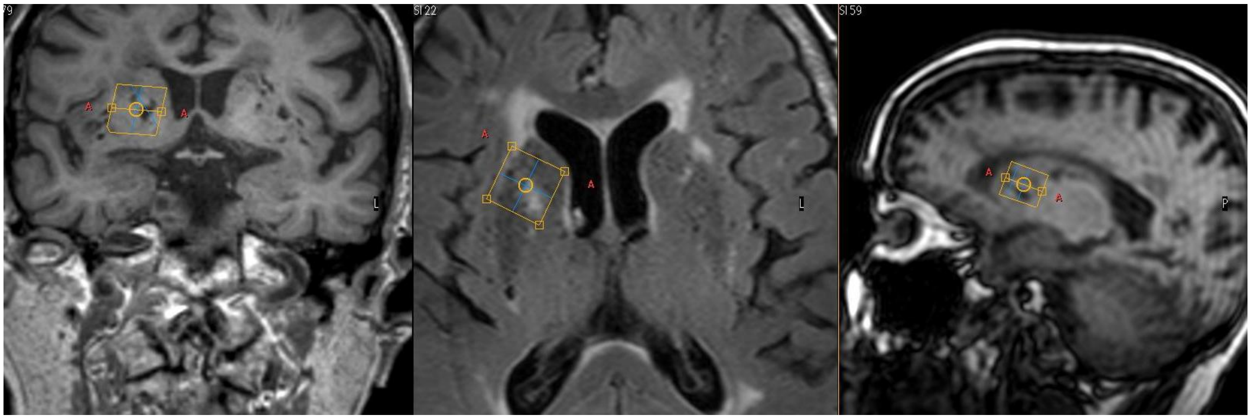

Rationale for voxel selection

The frontal white matter has been systematically studied in the cART era neuroHIV studies and abnormalities related to age and HIV infection have been found (see introduction). The caudate nucleus was selected rather than other basal ganglia nuclei based on a review by Paul et al. [15] which has identified that the caudate nuclei are a primary target of HIV infection. The posterior cingulate cortex has been systematically used in normal aging and pathological aging studies (see introduction).

Rationale for unsuppressed water signal (H_2O) as the main reference

We used the unsuppressed water signal (H_2O) as the main reference rather than Creatine. There is now cumulative evidence that Creatine is not constant across brain region including in non-pathological state such as normal aging for example. The use of H_2O as the reference marker is now advocated as an alternative except when water variations are expected in certain clinical

conditions. To gain specific understanding on the use of H_2O as the reference and the associated MRS protocols we recommend the textbook by Barker, et al. [16].

Spectra illustrations

Frontal white matter

HIV+ case

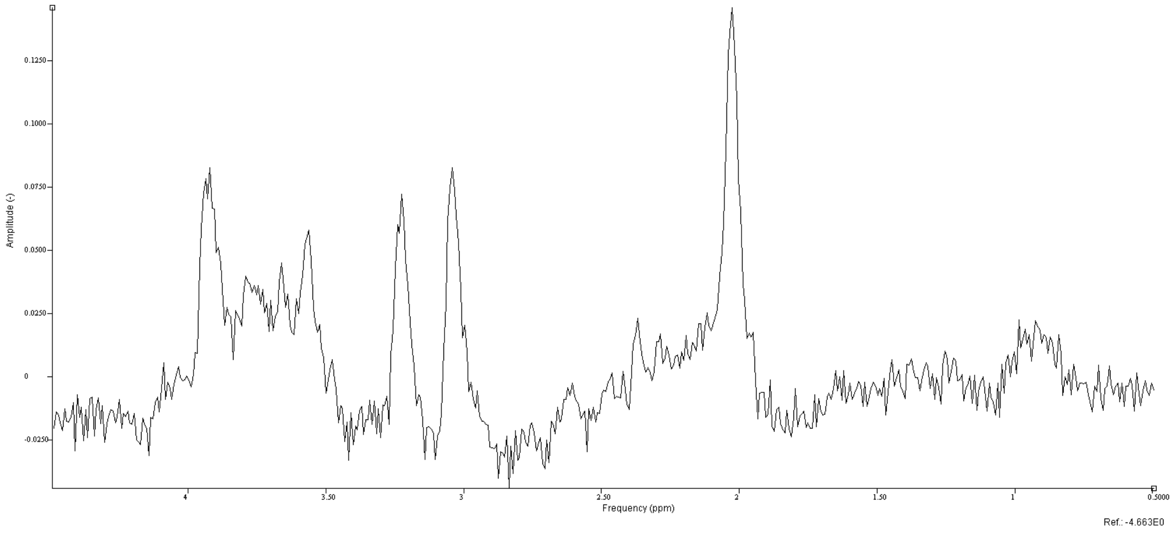

Frequency (ppm)
- NAA: 2.028
- Cr: 3.039
- Cho: 3.228
- mIo: 3.569
- Glx: 2.281 ~ 2.37

Linewidth (Hz)
- NAA: 6.54
- Cr: 6.76
- Cho: 5.28
- mIo: 6.76
- Glx: 8.5 ~ 6.5

HIV- case

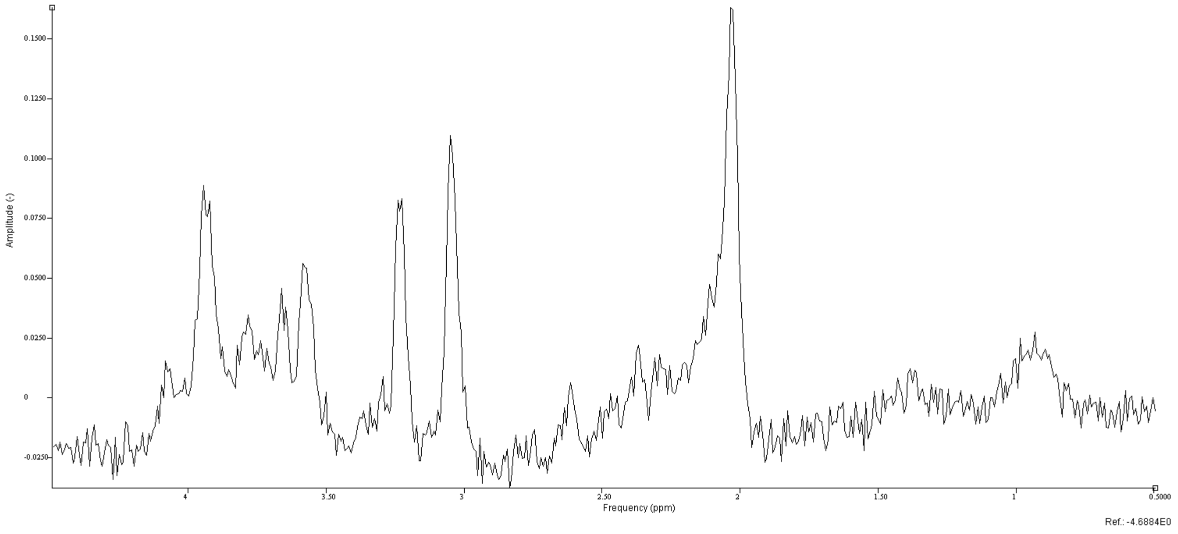

Frequency (ppm)
- NAA: 2.019
- Cr: 3.03
- Cho: 3.22
- mIo: 3.558
- Glx: 2.28 ~ 2.36

Linewidth (Hz)
- NAA: 6.54
- Cr: 6.76
- Cho: 5.28
- mIo: 6.76
- Glx: 8.5 ~ 6.5

Posterior Cingulate Cortex

HIV+ case

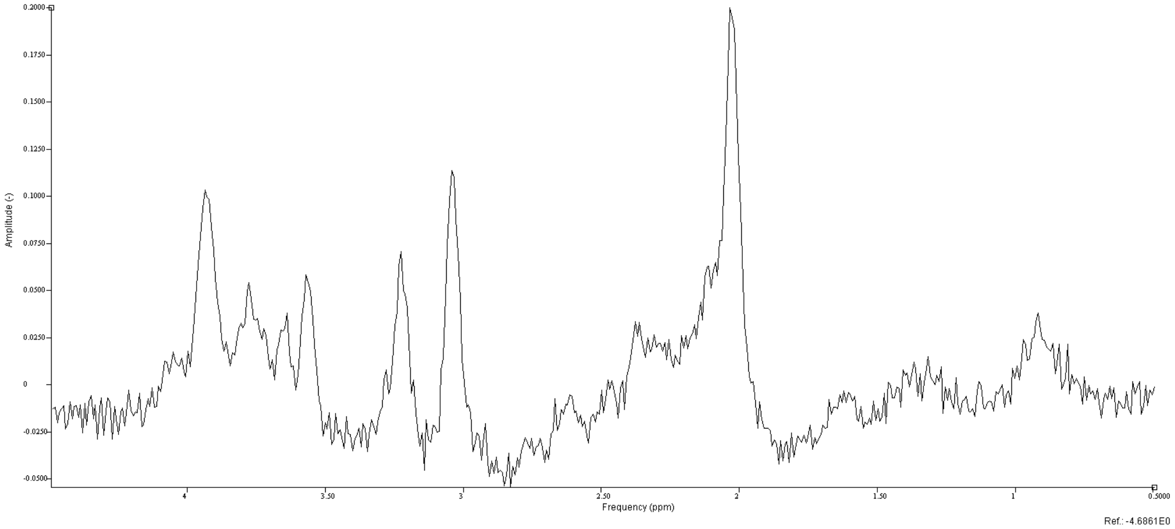

Frequency (ppm)
- NAA: 2.01
- Cr: 3.022
- Cho: 3.209
- mIo: 3.549
- Glx: 3.916

Linewidth (Hz)
- NAA: 6.54
- Cr: 6.76
- Cho: 5.28
- mIo: 6.76
- Glx: 8.5 ~ 7.5

HIV- case

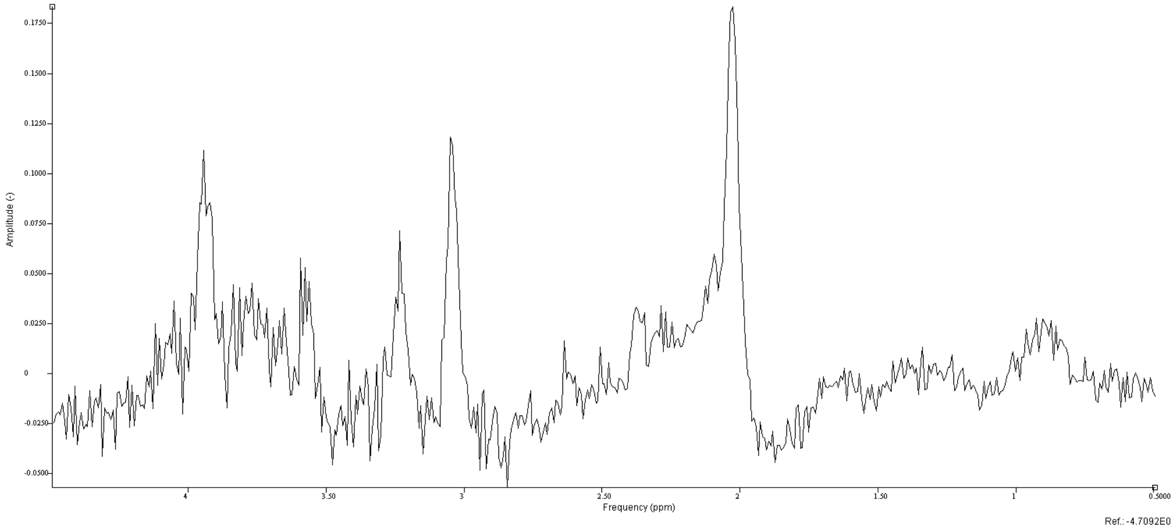

Frequency (ppm)
- NAA: 2.026
- Cr: 3.041
- Cho: 3.230
- mIo: 3.569
- Glx: 2.28 ~ 2.36

Linewidth (Hz)
- NAA: 6.54
- Cr: 6.76
- Cho: 5.28
- mIo: 6.76
- Glx: 8.5 ~ 8.01

Caudate nucleus area

HIV+ case

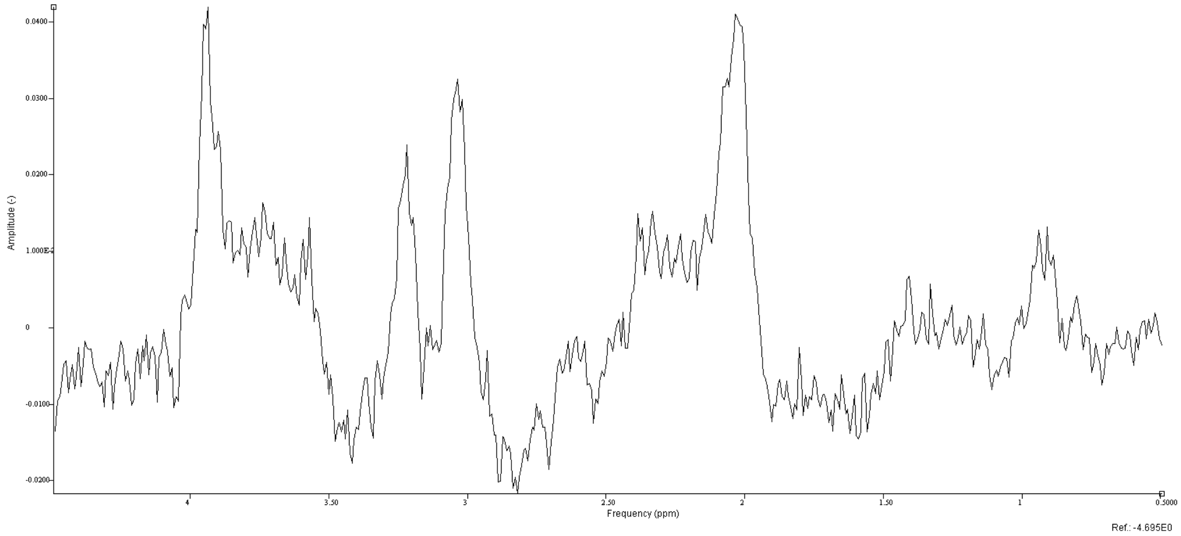

Frequency (ppm)
- NAA: 2.03
- Cr: 3.036
- Cho: 3.222
- mIo: 3.569

Linewidth (Hz)
- NAA: 7.0
- Cr: 7.0
- Cho: 6.22
- mIo: 8.0

HIV- case

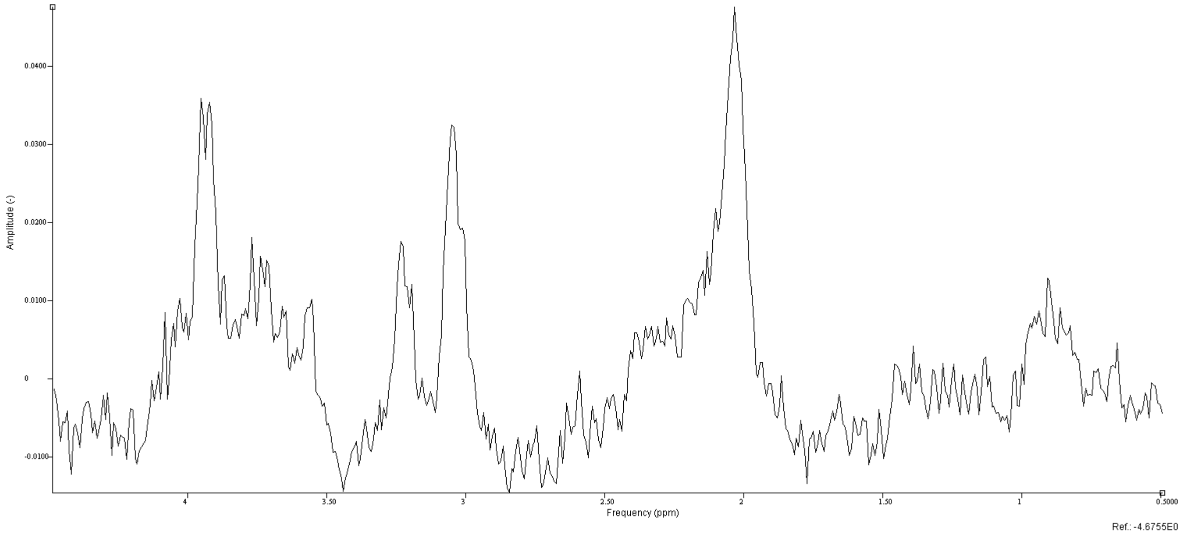

Frequency (ppm)
- NAA: 2.023
- Cr: 3.035
- Cho: 3.215
- mIo: 3.569

Linewidth (Hz)
- NAA: 7.0
- Cr: 7.0
- Cho: 6.3
- mIo: 8.0

Figure S1: Frontal White Matter Cr/H_20 and mIo/ H_20 in the HIV- and HIV+ groups

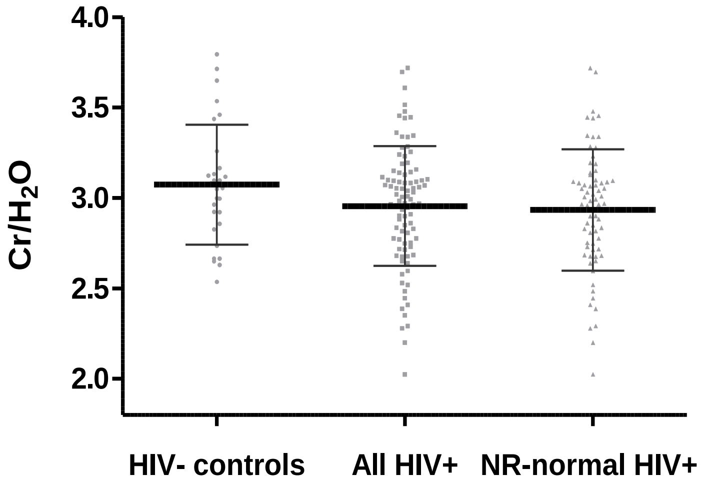

HIV- vs. all HIV+: p<.10; d=.36; vs. Neurocognitively-normal (NR) HIV+: *p*<.07

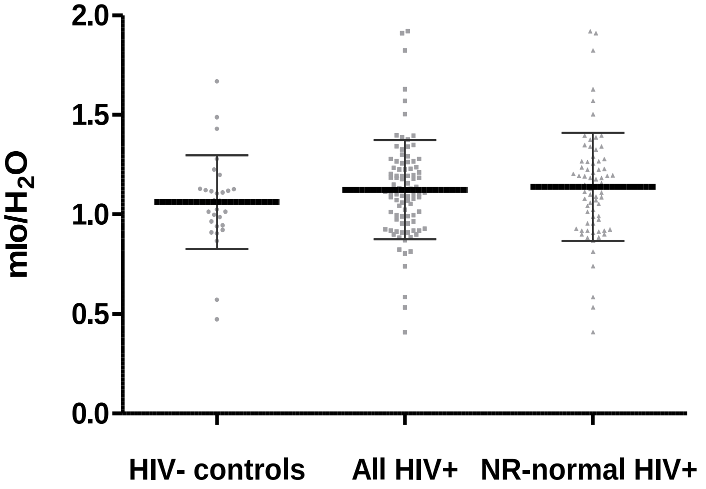

HIV- vs. all HIV+: p<.22; d=.26; vs. Neurocognitively-normal (NR) HIV+: *p*<.17

Figure S2: Posterior Cingulate Cortex NAA/H_20 and mIo/ H_20 in the HIV- and HIV+ groups

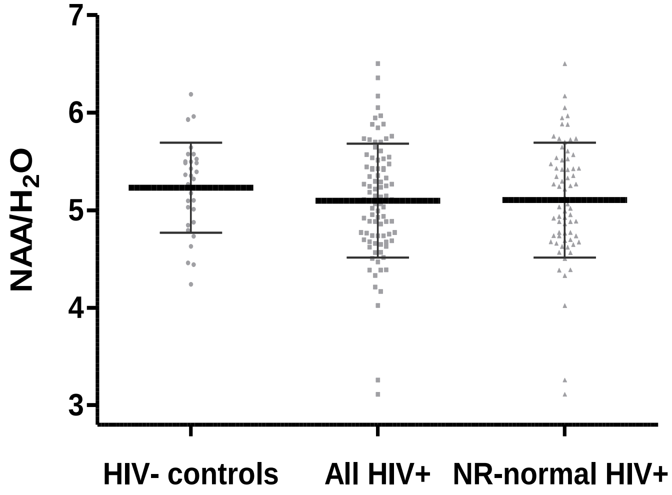

HIV- vs. all HIV+: p<.20; d=.026; vs.Neurocognitively-normal (NR) HIV+: *p*<.25

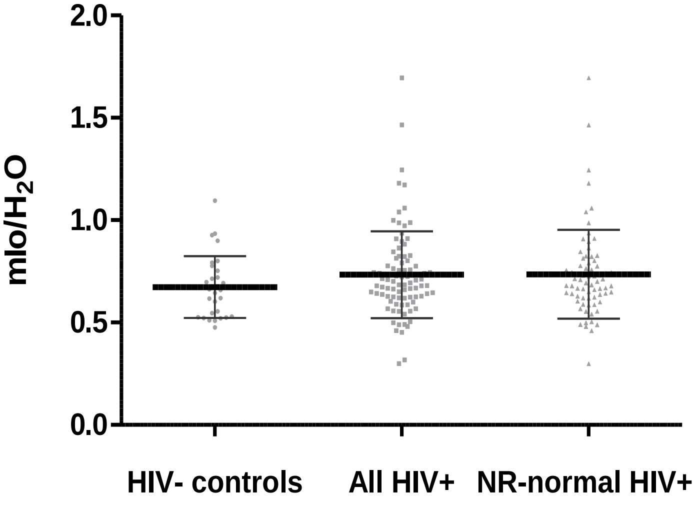

HIV- vs. all HIV+: p<.09; d=.30; vs. Neurocognitively-normal (NR) HIV+: *p*<.05

Figure S3: Caudate Nucleus Area Cr/$H_2$0 in the HIV- and HIV+ groups

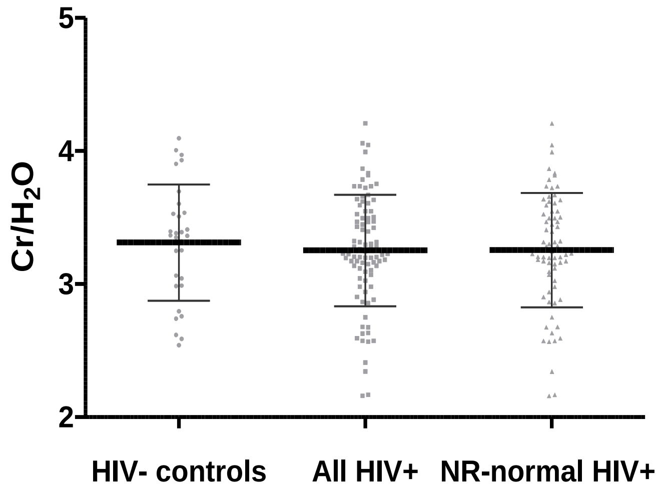

HIV- vs. all HIV+: p>.50; d=.14; vs. Neurocognitively-normal (NR) HIV+: *p*>.54

File S3

Tonometry examination procedure

HIV+ participants underwent non-invasive tonometry to estimate arterial stiffness of the radial artery. Pulse pressure was used as research indicated that it is a better predictor of cardiac ischemic events than systolic, diastolic, or mean brachial pressures [17]. The evaluation was performed in duplicate by a single operator at the left radial artery using a highly sensitive transducer (SphygmoCor, AtCor Medical, Sydney). An augmentation index was calculated by dividing the difference between the second systolic peak and the diastolic pressure by the difference between the first systolic peak and the diastolic pressure and expressed as a percentage. Systolic blood pressure was also recorded at this time.

Extra Results section

Neurocognitive and mood and functional characteristics in study groups

The HIV+ individuals as a group showed a significantly greater degree of overall neurocognitive impairment (Table S2). We found that 23% of HIV+ participants had clinically relevant (GDS≥0.5) level of neuropsychological (NP) deficits or HAND. Cognitive complaints were significantly more frequent in the HIV+ group than the HIV- group. While the HIV+ group also had a mild level of depressive complaints, the HIV+ individuals who reported clinically significant levels of depressive complaints (16%; BDI-II total score > 17) were no more likely to be NP impaired than those who did not (p>.27). Almost a quarter of the HIV+ cohort self-reported a decrease in independence in activities of daily living. Furthermore, those who reported clinically significant decline activities of daily living were more likely to have HAND as

compared to those who did not report any decline (37% vs. 16%; *p*<.02). Also, those who reported functional decline and were not impaired were more likely to be depressed at a clinical level (*p*<.0001).

Single CVD laboratory markers, Tonometry Augmentation Index, and moieties concentrations results in the HIV+ group

Using Pearson correlations, a higher total cholesterol was significantly correlated with lower NAA in the posterior cingulate cortex (*r*=-.30; *p*<.005); a higher HDL cholesterol with a lower mIo in the frontal white matter (*r*=-.19; *p*=.05), a higher systolic blood pressure with lower NAA in the posterior cingulate cortex (*r*=-.29; *p*<.008). A lower NAA in the caudate nucleus and a higher tonometry Augmentation Index trended to significance (*r*=-.19; *p*<.07). The greater length of use of lopinavir/ritonavir was associated with lower NAA in the posterior cingulate (*r*=-.24; *p*<.03). Finally individuals with a high CRP (>4.7) had significantly lower NAA in the posterior cingulate (means of 4.8 vs 5.2; *p*<.02).

Table S2: Neurocognitive performance, Independence in Activities of Daily Living, and cognitive complaints in the HIV– and HIV+ groups

	HIV –	HIV+	*P*
Global Mean T-score	52.38 (3.98)	49.13 (6.77)	<.002
GDS	0.14 (0.18)	0.33 (0.62)	.009
Overall Neurocognitive impairment (GDS≥0.5)	3% (1/30)	22% (20/92)	.02
IADL summary score	0.16 (0.38)	1.23 (1.97)	<.0001
% IADL significant decline [1]	0%	26%	<.002
ANI / MND / HAD [1]	13% / 0% / 0%	10.8% / 3.3% / 7.6%	-
Cognitive Complaints PAOFI [2]	1.66 (2.08)	4.21 (4.83)	.0001
BDI-II total score [3]	3.27 (4.19)	9.02 (8.56)	<.0001
Clinically significant depression [4]	0%	16%	<.02

1. IADL significant change: Independence in Activities of Daily Living: self-report of at least 2 or more decrease in the capacity to perform IADL. The HAND criteria definition follows the [43] nomenclature and uses the IADL cut-off as previously defined to discriminate between ANI and MND. Note that the level of impairment in controls according to this nomenclature is close to be expected 15% when at least five cognitive domains have been measured. Also note that the GDS: Global Deficit Score dichotomous score and the HAND criteria provides same level of impairment in the HIV+ group, but not in the HIV- group.

2. PAOFI: Personal Assessment of Own Functioning Inventory total score
3. BDI-II: Beck Depression Inventory-II total score.
4. BDI-II clinically significant: A cut off of >17 was used to define clinically significant levels of depression.
Note that there was no significant correlation between the CVD scores and the global neuropsychological performance.

References

1. Beck AT, Steer RA, Ball R, Ranieri W (1996) Comparison of Beck Depression Inventories -IA and -II in psychiatric outpatients. J Pers Assess 67: 588-597.

2. Beck AT, Steer RA, Brown GK (1996) Manual for the Beck Depression Inventory-II. San Antonio: TX: Psychological Corporation.

3. Chelune G, Heaton R, Lehman R (1986) Neuropsychological and personality correlates of patient's complaints of disability. In: Tarter R, Goldstein G, editors. Advances in Clinical Neuropsychology. New York: New York Plenum Press. pp. 95-126.

4. Heaton RK, Marcotte TD, Mindt MR, Sadek J, Moore DJ, et al. (2004) The impact of HIV-associated neuropsychological impairment on everyday functioning. J Int Neuropsychol Soc 10: 317-331.

5. Simoni JM, Kurth AE, Pearson CR, Pantalone DW, Merrill JO, et al. (2006) Self-report measures of antiretroviral therapy adherence: A review with recommendations for HIV research and clinical management. AIDS Behav 10: 227-245.

6. Sheehan DV, Lecrubier Y, Sheehan KH, Amorim P, Janavs J, et al. (1998) The Mini-International Neuropsychiatric Interview (M.I.N.I.): the development and validation of a structured diagnostic psychiatric interview for DSM-IV and ICD-10. J Clin Psychiatry 59 Suppl 20: 22-33;quiz 34-57.

7. Brandt J, Benedict RH (2001) Hopkins Verbal Learning Test-Revised. Professional manual. Lutz, FL: Psychological Assessment Resources, Inc.

8. Heaton RK, Miller SW, Taylor MJ, Grant I (2004) Revised comprehensive norms for an expanded Halstead-Reitan Battery: Demographically adjusted neuropsychological norms for

African American and Caucasian adults Scoring Program. Odessa: FL: Psychological Assessment Ressources.

9. Carey CL, Woods SP, Gonzalez R, Conover E, Marcotte TD, et al. (2004) Predictive validity of global deficit scores in detecting neuropsychological impairment in HIV infection. J Clin Exp Neuropsychol 26: 307-319.

10. Klove H (1963) Clinical Neuropsychology. In: Forster FM, editor. The Medical Clinics of North America. New York: Saunders.

11. Wechsler D (1997) Wechsler Adult Intelligence Scale-Third Edition manual. San Antonio: The Psychological Corporation.

12. Wechsler D (1997) Wechsler Memory Scale - Third Edition manual. San Antonio: The Psychological Corporation.

13. Delis DC, Kaplan E, Kramer JH (2001) Delis Kaplan Executive Function System (DKEFS). San Antonio, TX: The Psychological Corporation.

14. Benton AL, Hamsher K, Sivan AB (1994) Multilingual Aphasia Examination. Iowa City: AJA Associates.

15. Paul R, Cohen R, Navia B, Tashima K (2002) Relationships between cognition and structural neuroimaging findings in adults with human immunodeficiency virus type-1. Neurosci Biobehav Rev 26: 353-359.

16. Barker, P., Bizzi A., De Stefano N., Gullapalli R., Lin D. D. M., 2010, Clinical MR Spectroscopy: Techniques and Applications, Cambrigde University Press.

17. O'Rourke MF, Adji A. An updated clinical primer on large artery mechanics: implications of pulse waveform analysis and arterial tonometry. *Curr Opin Cardiol.* Jul 2005;20(4):275-281.
